# Transcranial Doppler during the first week after cardiac arrest and association with 6-month outcomes

**DOI:** 10.3389/fneur.2023.1222401

**Published:** 2023-10-04

**Authors:** Antje Reichenbach, Lars Alteheld, Julia Henriksen, Espen Rostrup Nakstad, Geir Øystein Andersen, Kjetil Sunde, Jūratė Šaltytė Benth, Christofer Lundqvist

**Affiliations:** ^1^Department of Neurology, Akershus University Hospital, Lørenskog, Norway; ^2^Department of Neurology, Oslo University Hospital Ullevaal, Oslo, Norway; ^3^Department of Acute Medicine, Oslo University Hospital Ullevaal, Oslo, Norway; ^4^Norwegian National Unit for Chemical, Biological, Radioactive, Nuclear, and Explosive Medicine, Oslo University Hospital Ullevaal, Oslo, Norway; ^5^Department of Cardiology, Oslo University Hospital Ullevaal, Oslo, Norway; ^6^Faculty of Medicine, Institute of Clinical Medicine, University of Oslo, Oslo, Norway; ^7^Department of Anesthesia and Intensive Care Medicine, Oslo University Hospital Ullevaal, Oslo, Norway; ^8^Health Services Research Unit, Akershus University Hospital, Lørenskog, Norway; ^9^Faculty of Medicine, Institute of Clinical Medicine, Campus Akershus University Hospital, University of Oslo, Oslo, Norway

**Keywords:** ultrasound, heart, out of hospital arrest, cardiac arrest (CA), brain, brain damage

## Abstract

**Background:**

Early prediction of outcomes in comatose patients after out-of-hospital cardiac arrest is challenging. Prognostication tools include clinical examination, biomarkers, and neuroradiological and neurophysiological tests. We studied the association between transcranial Doppler (TCD) and the outcome.

**Methods:**

This was a pre-defined sub-study of the prospective observational Norwegian Cardiorespiratory Arrest Study. Patients underwent standardized post-resuscitation care, including target temperature management (TTM) to 33°C for 24 h. TCD was performed at days 1, 3, and 5–7. The primary endpoint was cerebral performance category (CPC) at 6 months, dichotomized into good (CPC 1–2) and poor (CPC 3–5) outcomes. We used linear mixed modeling time-series analysis.

**Results:**

Of 139 TCD-examined patients, 81 (58%) had good outcomes. Peak systolic velocity in the middle cerebral artery (PSV) was low during TTM (Day 1) and elevated after rewarming (Day 3). Thereafter, it continued to rise in patients with poor, but normalized in patients with good, outcomes. At days 5–7, PSV was 1.0 m/s (95% CI 0.9; 1.0) in patients with good outcomes and 1.3 m/s (95% CI 1.1; 1.4) in patients with poor outcomes (p < 0.001)

**Conclusion:**

Elevated PSV at days 5–7 indicated poor outcomes. Our findings suggest that serial TCD examinations during the first week after cardiorespiratory arrest may improve our understanding of serious brain injury.

## Introduction

Cardiac arrest is a major cause of death, with an incidence rate up to 86 per 100,000 individuals in Europe (1, 2). Despite the return of spontaneous circulation after cardiopulmonary resuscitation and improved post-resuscitation care over the years, many patients remain unconscious for several days or weeks. ICU decisions regarding continued, limited, or terminated therapy carry huge ethical and socio-economic implications. Current guidelines for outcome prediction recommend a multimodal approach using clinical neurological examination, neurophysiological tests, biochemical parameters, and neuroimaging studies (3). A high specificity of prognostic methods is important to avoid false pessimistic evaluations of poor outcome risking self-fulfilling prophecies through the withdrawal of life-sustaining therapy (WLST).

Transcranial Doppler (TCD) is a simple and low-cost bedside examination that provides information about hemodynamic parameters in the large intracerebral arteries. It is widely used at neuro-intensive care units for non-invasive examination and monitoring of systolic blood flow velocity (PSV), diastolic velocity (DV), mean velocity (MV), pulsatility index (PI), and resistance index (RI) in patients with subarachnoidal hemorrhage, traumatic brain injury, and stroke.

TCD studies in patients after out-of-hospital cardiac arrest (OHCA) are rare, include few patients, often <50, and have a narrow time frame, often within the first 72 h after OHCA. Almost all of these TCD studies describe an initial period of cerebral hypoperfusion up to 24 h after OHCA followed by a hyperperfusion phase (4–7). Some studies found no correlation between TCD parameters and clinical outcomes (4–6), whereas others report conflicting results (7–10).

Based on these findings, we intended to study TCD parameters after OHCA on a larger cohort and over a longer time period. We aimed to test to what extent clinical outcome after 6 months was associated with i) bedside TCD parameters during cooling, immediately after rewarming and 5–7 days after OHCA, and ii) time-dependent patterns of serial TCD examinations over the 1st week after OHCA.

## Methods

The Norwegian Cardiorespiratory Arrest Study (NORCAST, NCT 01239420) was a prospective observational study performed at Oslo University Hospital Ullevål, where 259 adult comatose OHCA patients were included between 2010 and 2014. The overall aim was to provide physicians with better tools to assess cardiac and neurological outcomes as early and accurately as possible. All patients underwent standardized post-resuscitation care, including targeted temperature management (TTM) to 33°C for 24 h. Rewarming started 24 h after admittance, with 0.5°C/h temperature increase. Normothermia was reached on day 2. All study assessments were blinded to clinical information and performed independently from the treating staff. The treating physicians continued to use established treatment paradigms which at this time emphasized no calls for hasty outcome prediction or early WLST, and a multidisciplinary clinical and neurological assessment with only the occasional use of neuron-specific enolase (NSE), somatosensory evoked potentials, and electroencephalography. After 6 months, 49% of the patients were alive with a good outcome (CPC 1–2). Time to awakening in patients with good outcomes was a median of 6 days (11).

### TCD measurements

In the present pre-planned, prospective sub-study, bedside TCD blinded to the treating physician was performed three times during the first week: on days 5–7 (124–168 h) as pre-planned and additionally when possible also on day 1 (within 24 h during TTM) and day 3 (within 48–72 h after TTM). All ultrasound examinations were performed by one of two experienced investigators (AR or LA) using a transcranial 2.0 MHz pulsed probe of a color-coded duplex ultrasound system (Philips HD11xe or Philips iE33 depending on which machine was available). Though no formal inter-rater variability was assessed, the two ultrasound investigators had been working closely together for several years, which should ensure an acceptable inter-rater variability.

**Figure 1:**
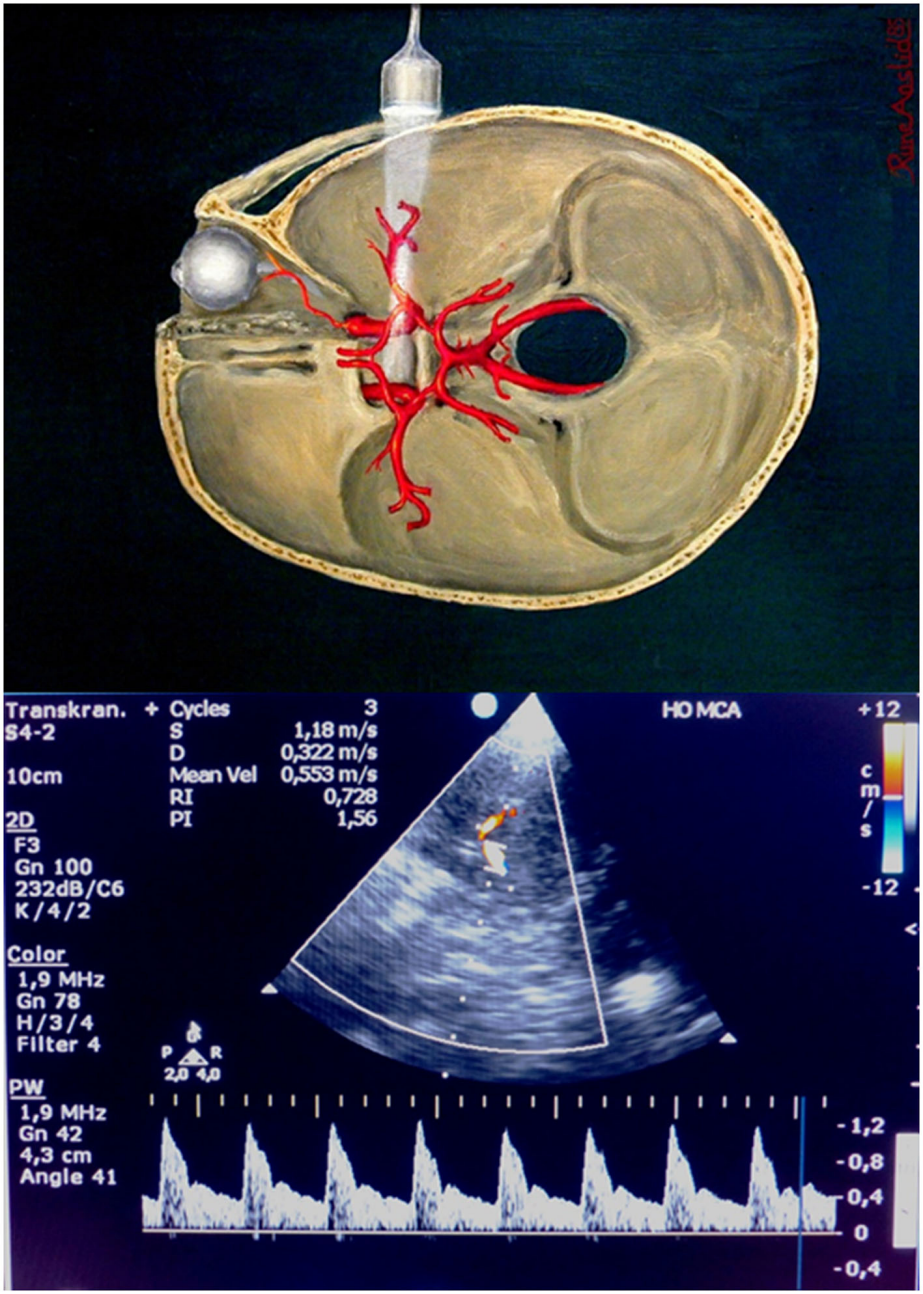
Bedside transcranial Doppler of the middle cerebral artery (the figure is intended to illustrate the basic principle and anatomy of transcranial ultrasound and may not show exactly the same probe as used in the study). (Figure above reproduced by kind permission from Rune Aaslid).

The following data were recorded: PSV, DV, MV, PI, and RI for the internal carotid arteries, the vertebral arteries, and the middle cerebral arteries (MCA) on both sides, body temperature, heart rate, and arterial blood pressure (Figure 1). Patients with stenotic lesions of hemodynamic relevance and patients without sufficient bone window were excluded. Velocity measurements in the MCA were performed without angle correction since the depicted vessel was usually short. Based on clinical experience and relevant literature, we defined a PSV cutoff of 1.1 m/s for elevated PSV (12, 13).

### Brain damage markers

To relate TCD parameters to neuronal cell damage, serum NSE and S100B values were measured at days 1, 3, and 5–7. NSE levels were analyzed using an in-house immunoradiometric assay with samples in which strong hemolysis was excluded. S100B levels were analyzed using a two-site sandwich immunoradiometric assay for the β-subunit [S100B, DiaSorin (formerly named Sangtec), Stillwater, Minnesota, USA]. The test was used according to the manufacturer's protocol.

### Main outcome

The primary outcome was the CPC score assessed 6 months after OHCA by two experienced neurologists, using standardized clinical examination (14). The CPC score of 1–2 was classified as a good outcome, and the CPC score of 3–5 was classified as a poor outcome.

### Statistics

Demographic and clinical data were presented as means and standard deviations (SDs) or frequencies and percentages. Patients with good and poor outcome were compared by the chi^2^ test for categorical variables and the independent sample *t*-test or Mann–Whitney U-test for continuous data. Differences in PSV at days 5–7 between good and poor outcome groups were assessed by the independent samples *t*-test. The discriminatory value of PSV with respect to outcomes was assessed by receiver operator characteristic curve analysis and area under the curve with the corresponding 95% confidence interval (CI). Sensitivity, specificity, positive predictive value, and negative predictive value were calculated for a pre-defined cutoff of 1.1 m/s. The predictive values for RI and PI were also assessed.

Time trend in TCD parameters (PSV, MV, NSE, and S100B) was assessed by linear mixed models with random effects for patients to account for within-patient correlations due to repeated measurements. Fixed effects for time points, outcome (good or poor), and the interaction between these two were included. A significant interaction would imply that the time trend is different in different outcome groups. *Post-hoc* analyses were performed to assess between-group differences at different time points. The results of *post-hoc* analyses were presented as mean differences with corresponding 95% CIs and *p*-values and are illustrated graphically. All tests were two-sided, and the results with *p*-values below 0.05 were considered statistically significant. Statistical analyses were performed in SPSS v26 and STATA v17.

### Ethical issues

The project was based on informed consent by patients or, in comatose patients, by next of kin as approved by the responsible research ethical committee in Norway (REK-S-O/A-2010/1116a). All data were stored and de-identified on a secure data platform as approved by the data inspectorate officer.

## Results

**Figure 2:**
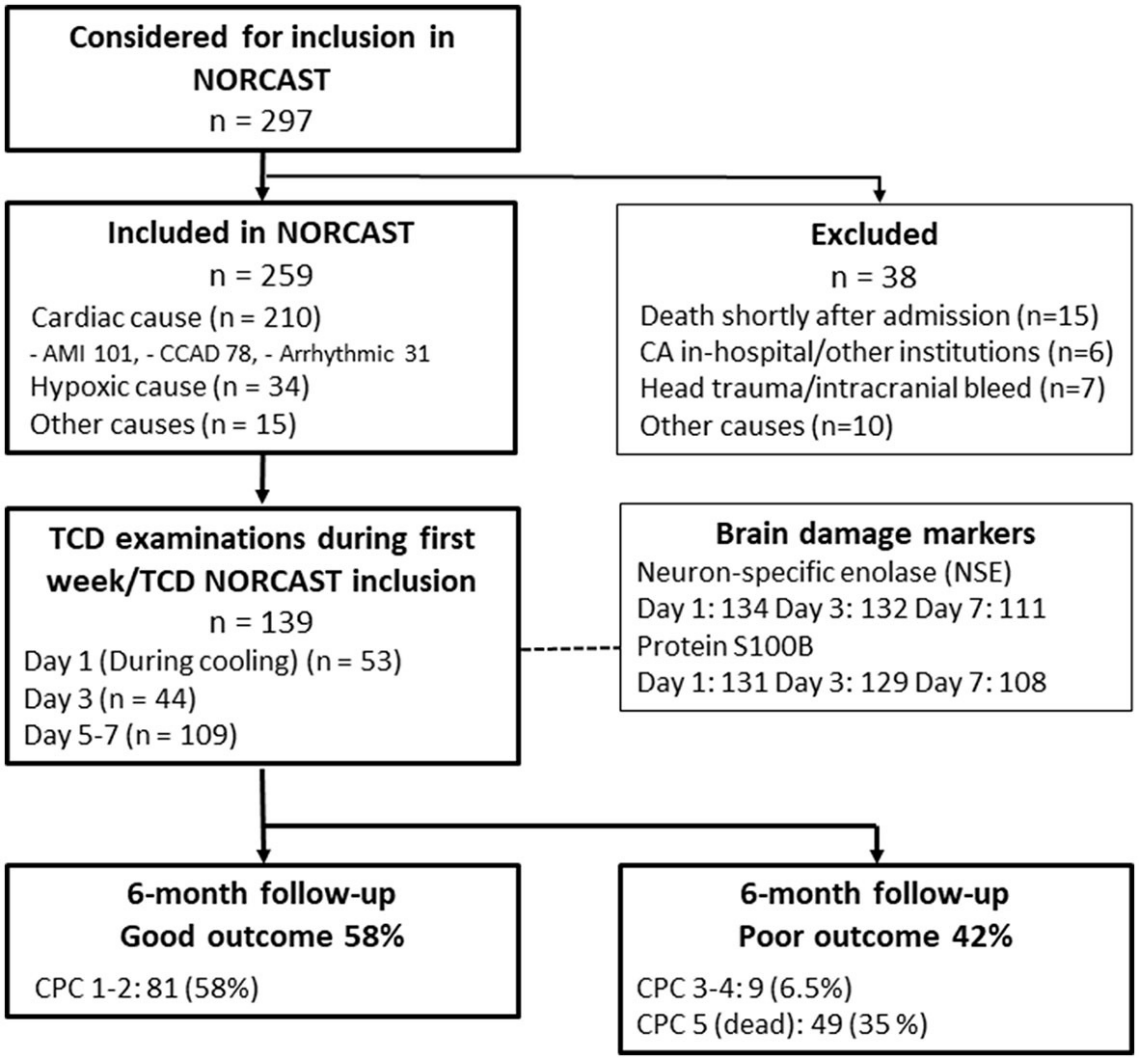
STROBE flowchart of patients included in the TCD sub-study. AMI, acute myocardial infarction; CCAD, chronic coronary artery disease; CA, cardiac arrest; CPC, cerebral performance category.

TCD data were collected from 139 out of 259 patients included in the NORCAST study (*n* = 53, *n* = 44 and *n* = 109 at days 1, 3, and 5–7, respectively). Mean body temperature on day 1 was 33.1°C (31.9; 34.5), mean temperature on day 3 was 37.5°C (36.1; 38.5), and mean temperature on days 5–7 was 37.6°C (36.7; 39.9). The intention was to include all possible patients at the mentioned time points but logistic challenges in a clinical setting, such as staff shortage or patients undergoing other examinations, were the main reasons for missing data during and early after TTM (day 1 and day 3). Early death or early transfer to other hospitals was the main reason for missing data on days 5–7. Of the 139 included patients, 81 patients (58%) had good outcomes (CPC 1–2) at 6 months. Figure 2 shows the number of patients included. Table 1 shows demographic and clinical data.

**Table 1:** Demographic and clinical data.

	**All (*n =* 139)**	**Good (*n =* 81)**	**Poor (*n =* 58)**	***p*-value (good vs. poor)**
Gender, M/F (%)	116/23 (83.5/16.5%)	70/11 (86.4/13.6%)	46/12 (79.3/20.3%)	0.27^a^
Age (SD)	60.2 (12.9)	58.7 (12.3)	62.3 (13.6)	0.11^b^
Heart rate days 5–7 (SD) (*n =* 104)	81.5 (17.5)	77.2 (15.1)	87.7 (19.1)	0.002^b^
Time to return of spontaneous circulation(SD) (*n =* 123)	26.5 (17.8)	23.2 (17.2)	31.7 (17.7)	0.001^c^
**Cause of arrest**				
Ischemic	99 (71.2%)	59 (72.9%)	40 (69%)	0.008^a^
Arrhythmic	15 (10.8%)	14 (17.3%)	1 (1.7%)	
Hypoxia	18 (12.9%)	6 (7.3%)	12 (20.7%)	
Others/unknown	7 (5.1%)	2 (2.4%)	5 (8.6%)	
**Cause of death**				
Cardial/Prob.card.	6 (4.3%)	0	6 (4.3%)	Not relevant
CerebralProb.cer.	36 (26.9%)	0	36 (26.9%)	
Others/unknown	7 (5.0%)	0	7 (5.0%)	
**Initial rhythm**				
VF/VT	101 (72.7%)	69 (85.2%)	32 (55.1%)	0.003^a^
Asystole	22 (15.8%)	6 (7.4%)	16 (27.6%)	
PEA	13 (9.4%)	4 (4.9%)	9 (15.5%)	
Sinusbrady/unknown	3 (2.1%)	2 (2.4%)	1 (1.7%)	

a chi^2^-test.

b *t*-test.

c Mann–Whitney U-test.

SD, standard deviation.

VF/VT, ventricular fibrillation/ventricular tachycardia; PEA, pulseless electrical activity.

### Relationship between TCD parameters and outcome

**Figure 3:**
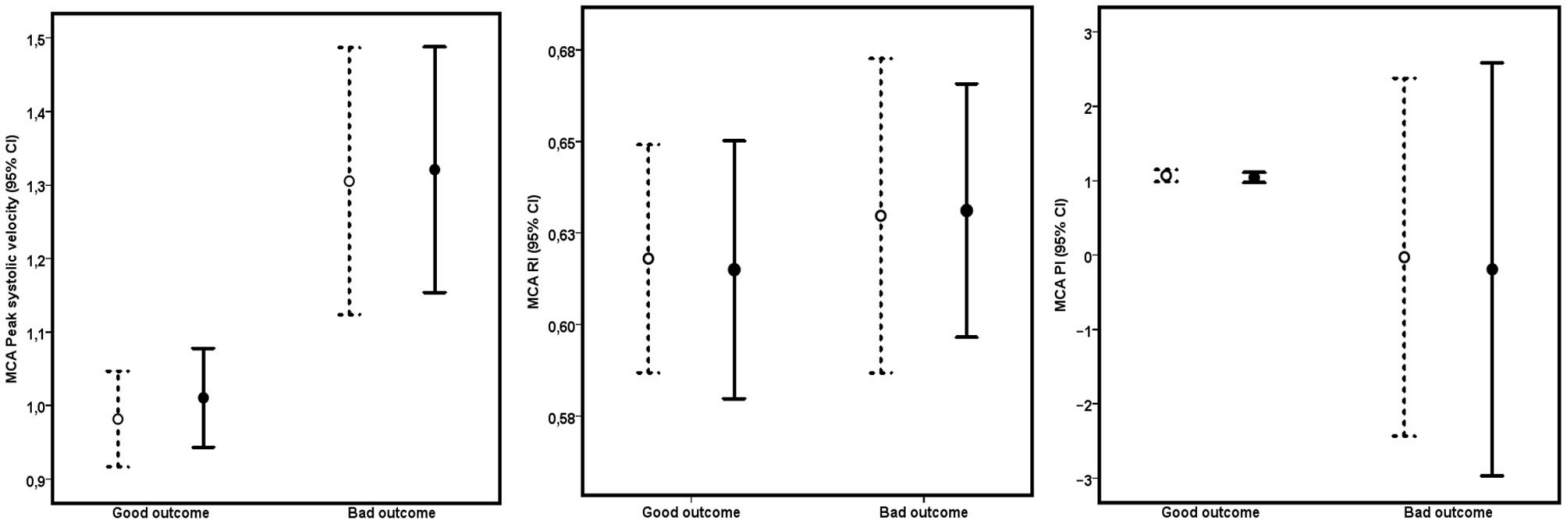
Relationship between day 7 TCD parameters (PSV, PI, and RI in the right (open symbols, dashed lines) and left (filled symbols, continuous lines) MCA and clinical outcome. Numbers are means and 95% confidence intervals.

There was a significant relationship between MCA PSV at days 5–7 and outcome at 6 months (*p* = 0.003, Figure 3). Patients with good outcomes had significantly lower PSV in the middle cerebral artery [1.0 m/s, 95% CI (0.9; 1.0)] than patients with poor outcomes [1.3 m/s, 95% CI (1.1; 1.4)]. Receiver operator characteristic analyses for PSV at days 5–7 (unadjusted) to predict poor outcomes showed an area under the curve of 0.69 [95%CI (0.58–0.80)]. Using a cutoff value of 1.1 m/s resulted in sensitivity and specificity of 0.61 and 0.70, respectively, with a positive predictive value of 0.57 and a negative predictive value of 0.73. PI and RI at days 5–7 were not significantly associated with the dichotomous outcome.

**Figure 4:**
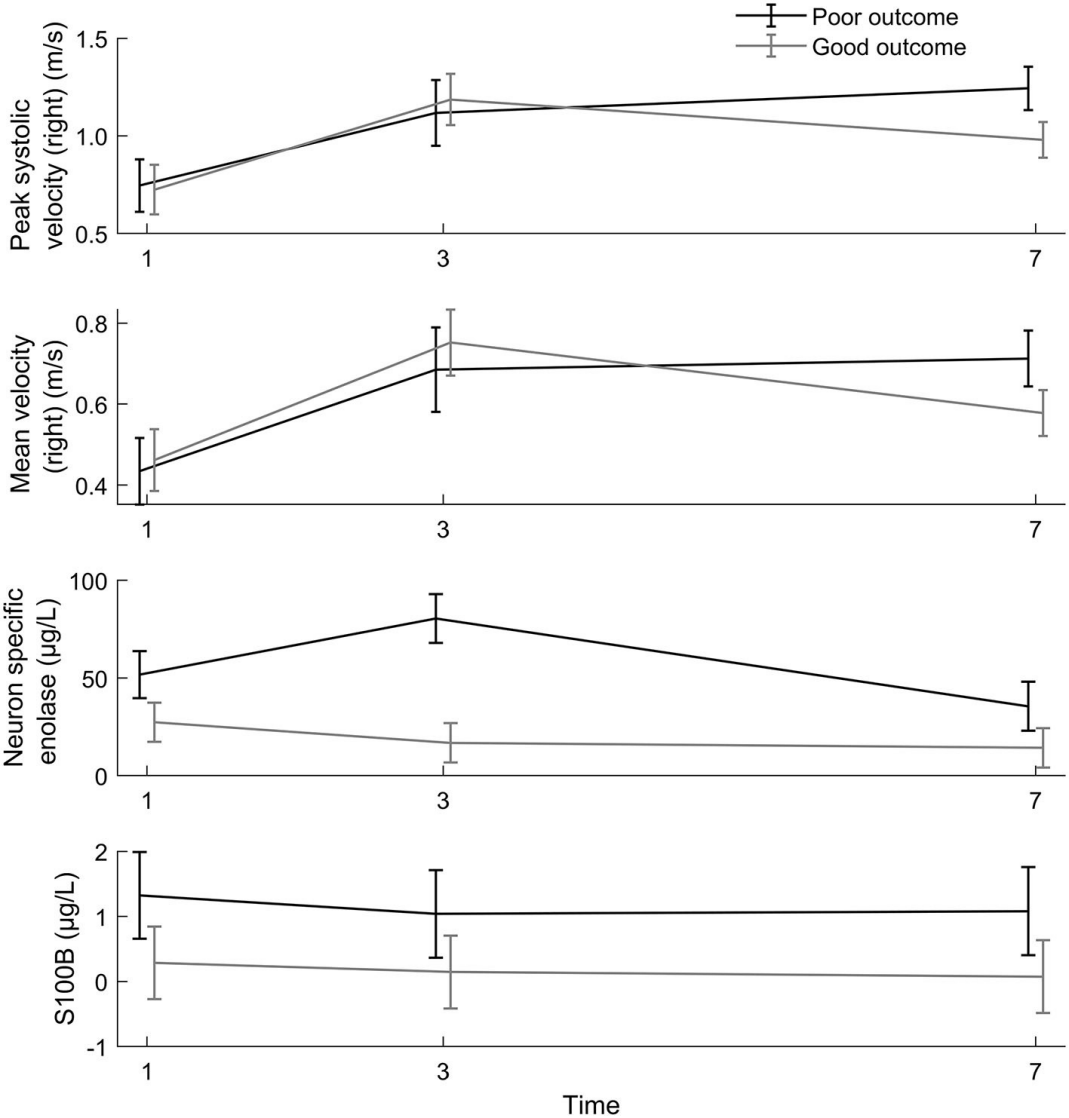
Time trend in PSV and MV, results of the unadjusted linear mixed model. The linear mixed model showed a significant time dependence (*p* = 0.004) and a significant difference in flow pattern between outcome groups (*p* < 0.001), see also Table 2.

According to linear mixed model analyses, PSV and MV in the middle cerebral artery showed distinct time-dependent dynamics, whereas flow velocities were low during TTM and elevated straight after rewarming in all patients, and they continued to rise within the first week in patients with poor outcomes but normalized in patients with good outcomes (Table 2, Figure 4). Table 2 and Figure 4 also illustrate the serial measurements of the brain damage markers NSE and S100B which also differed significantly between the outcome groups albeit with the expected differential time profile based on the different half-lives in serum of these two markers.

**Table 2:** Results of linear mixed models for PSV and MV in MCA (right side data shown, left side data with similar results).

**Parameter**	**Unadjusted model**		**Model adjusted for age and gender**	
	**Regr. coeff. (SE)**	**p-value**	**Regr. coeff. (SE)**	**p-value**
**PSV (*****N*** = **52 at Day 1**, ***N*** = **44 at Day 3**, ***N*** = **109 at Days 5–7)**				
Intercept	0.98 (0.05)	< 0.001	1.19 (0.14)	< 0.001
Day 1	−0.25 (0.06)	< 0.001	−0.25 (0.06)	< 0.001
Day 3	0.21 (0.07)	0.004	0.21 (0.07)	0.004
Day 5-7 (ref)	0		0	
CPC outcome	0.26 (0.07)	< 0.001	0.28 (0.07)	< 0.001
CPC outcome x Day 1	−0.24 (0.09)	0.014	−0.25 (0.09)	0.012
CPC outcome x Day 3	−0.33 (0.11)	0.004	−0.33 (0.12)	0.006
CPC outcome x Day 5-7	0		0	
**MV (*****N** =* **53 at Day 1**, ***N** =* **44 at Day 3**, ***N** =* **109 at Days 5–7)**				
Intercept	0.58 (0.03)	< 0.001	0.72 (0.09)	< 0.001
Day 1	−0.12 (0.04)	0.003	−0.11 (0.04)	0.005
Day 3	0.17 (0.04)	< 0.001	0.17 (0.04)	< 0.001
Day 5-7 (ref)	0		0	
CPC outcome	0.13 (0.05)	0.004	0.14 (0.05)	0.002
CPC outcome x Day 1	−0.16 (0.06)	0.006	−0.16 (0.06)	0.005
CPC outcome x Day 3	−0.20 (0.07)	0.005	−0.19 (0.07)	0.008
CPC outcome x Day 5–7	0		0	
**NSE (*****N** =* **134 at day 1**, ***N** =* **132 at day 3**, ***N** =* **111 at days 5–7)**				
Intercept	14.23 (5.12)	0.006	27.96 (11.41)	0.015
Day 1	13.15 (2.23)	< 0.001	13.40 (2.26)	< 0.001
Day 3	2.56 (6.17)	0.679	2.57 (6.17)	0.678
Day 7 (ref)	0		0	
CPC outcome	21.32 (8.18)	0.010	22.14 (8.20)	0.007
CPC outcome x Day 1	3.03 (3.91)	0.441	3.04 (3.99)	0.448
CPC outcome x Day 3	42.38 (9.91)	< 0.001	42.47 (9.92)	< 0.001
CPC outcome x Day 7 (ref)	0		0	
**S100 (*****N** =* **131 at day 1**, ***N** =* **129 at day 3**, ***N** =* **108 at days 5–7)**				
Intercept	0.08 (0.28)	0.792	0.08 (0.52)	0.885
Day 1	0.21 (0.08)	0.007	0.21 (0.08)	0.007
Day 3	0.07 (0.14)	0.610	0.07 (0.14)	0.610
Day 7 (ref)	0		0	
CPC outcome	1.00 (0.45)	0.027	1.00 (0.45)	0.028
CPC outcome x Day 1	0.03 (0.13)	0.820	0.03 (0.14)	0.799
CPC outcome x Day 3	−0.11 (0.22)	0.621	−0.11 (0.22) 0	0.627
CPC outcome x Day 7 (ref)	0		0	

According to *post-hoc* analyses (Table 3), PSV and MV at day 1 and day 3 were not associated with clinical outcomes at 6 months, whereas at days 5–7, they were age and gender-adjusted (*p* < 0.001 and *p* = 0.002, respectively).

**Table 3:** Results of *post-hoc* analyses of linear mixed models for PSV and MV in MCA, adjusted for age and gender (right side data shown, left side data with similar results).

	**Poor outcome Mean (95% CI)**	**Good outcome** **Mean (95% CI)**	**Mean difference (95% CI)**	**p-value**
**PSV**				
Day 1	0.97 (0.66; 1.29)	0.94 (0.64; 1.24)	0.03 (-0.15; 0.22)	0.720
Day 3	1.35 (1.01; 1.69)	1.39 (1.10; 1.69)	−0.04 (-0.26; 0.17)	0.690
Days 5–7	1.47 (1.17; 1.77)	1.19 (0.91; 1.46)	0.28 (0.14; 0.43)	< 0.001
**MV**				
Day 1	0.59 (0.39; 0.78)	0.61 (0.42; 0.79)	−0.02 (-0.13; 0.09)	0.725
Day 3	0.84 (0.63; 1.05)	0.89 (0.71; 1.07)	−0.05 (-0.129; 0.08)	0.462
Days 5–7	0.86 (0.67; 1.05)	0.72 (0.54; 0.89)	0.14 (0.05; 0.23)	0.002

Though not our primary focus, we also performed *post-hoc* analyses of the relationship between end-diastolic velocity (EDV) and clinical outcome at 6 months. The mean EDVs (right side) were 0.29 m/sec at day 1 (s.d. 0.16), 0.51 m/sec at day 3 (s.d. 0.23), and 0.41 m/sec at day 7 (s.d. 0.19). Significant differences (unadjusted) were found at day 7 with 0.46 (s.d.0.24) for those with poor outcomes and 0.38 (s.d.0.13) for good outcomes (*p* = 0.019).

## Discussion

Our main findings are that PSV and MV (and EDV) assessed through bedside TCD during the first week after OHCA are associated with clinical outcomes after 6 months. This is true both for single point measurements at days 5–7 and is supported by the time-dependent pattern of serial measurements over three different time points through the first week.

In accordance with previous studies (4–7), blood flow velocities were generally low during TTM and abnormally elevated straight after rewarming. However, we did not find TCD differences between the good and poor outcome groups within the first 72 h after OHCA although previous studies on smaller cohorts found both higher PI and higher RI values in patients with poor outcomes (8–10). Traditionally, both PI and RI have been used to describe distal cerebrovascular resistance, thus reflecting factors such as intracranial pressure (15, 16), but probably only in cases of markedly elevated pressure (17–20). Such extreme values may be more common among neurosurgical patients with subarachnoidal hemorrhage or traumatic brain injury but seem to be underrepresented in poor outcome patients of our study.

Our results clearly show that blood flow velocity abnormalities in the cerebral arteries are not confined to the first 72 h after OHCA but also occur later, at least up to 7 days. Between day 3 and days 5–7, the blood flow velocities continued to rise in patients with poor outcomes but normalized in patients with good outcomes. Longitudinal and single point measurements of MCA PSV at days 5–7 were associated with outcomes after 6 months, and patients with poor outcomes had significantly higher flow velocities at days 5–7 compared to those with good outcomes. Similar results have been described by Lovett et al. who followed 15 children with daily Doppler ultrasound for 8 days after a global hypoxic-ischemic event (21). Doepp et al., however, who performed serial TCD at days 1–2, days 3–5, and days 7–10 after OCHA, did not find such differences between good and poor outcomes, but this may also be explained by the very small number of patients with poor outcome still alive at days 7–10 (22).

Irreparable neuronal damage after cardiac arrest can be caused early and directly by the deprivation of blood and oxygen supply or delayed and indirectly driven by biochemical changes, reperfusion injury, and oxidative stress (18). In this late phase, much of the impaired blood flow in the brain occurs on the microcirculatory level not detectable directly by TCD. Blood flow velocities in the large cerebral arteries, however, are assumed to reflect general cerebral hypo- or hyperperfusion (22). Therefore, we propose that TCD parameters could be interpreted as an indirect sign of brain damage in the late phase. Our results suggest that an acceptable physiological hyperperfusion phase after rewarming extends toward days 2–3 but not beyond days 5–7. In case of recovery, cerebral perfusion would normalize within the first week after OHCA. Comprehensive microcirculatory damage could stimulate vasodilatation leading to ongoing hyperperfusion beyond 5–7 days, which may further increase late reperfusion injury.

The parallel assay of serial NSE and S100B in serum also showed corresponding differences between the two outcome groups, thus supporting the TCD measurements as being related to brain damage processes. The pattern of serial NSE measurements in the NORCAST study, commonly used as a central part of multimodal prognostication, has been presented and discussed elsewhere (11), and it is beyond the scope of the present manuscript to discuss these in detail here.

In summary, we postulate that blood flow velocities in the middle cerebral arteries at days 5–7 reflect cerebral macrocirculation changes secondary to microcirculation impairment. Ongoing cerebral hyperperfusion at the end of the first week after cardiac arrest may not, in itself, cause irreversible brain damage but may be interpreted as an indirect sign of ongoing brain injury, propagating the damaging process further. However, the pathophysiological mechanisms underlying our findings certainly need to be further elucidated. We hope our TCD studies can contribute toward raising further interest in this issue.

### Advantages and limitations

Compared to other TCD studies on this topic, important advantages in our study are the extended time window up to 7 days after cardiac arrest without a requirement for early withdrawal of treatment enabling us to follow most patients longer as well as the large and unselected sample. Nevertheless, there are some limitations that may have consequences for generalizability and should be considered in further studies.

We initiated the present study with the intention of doing TCD examinations on days 5–7 focusing on outcome prediction among still comatose OHCA patients but decided during the study period to include TCD at days 1 and 3 both to investigate cerebrovascular hemodynamics during and after TTM and the time-dependent pattern of serial TCD. Therefore, we have less day 1 and day 3 data (53 and 44 patients, respectively) compared to the number of days 5–7 data (109 patients), which may have contributed to the lack of association between early TCD and outcome after 6 months.

We collected our data between 2010 and 2014 when TTM to 32–34°C still was proposed. The latest guidelines, however, point out insufficient evidence for early cooling and recommend temperature control with a target between 32 and 36°C. Although a hypoperfusion period between 6 and 24 h after total cerebral ischemia is described in the case series already before the hypothermia era (18), the influence of temperature management on cerebral perfusion and especially on TCD parameters is not really known. Our patients had TTM33 (mean body temperature of 33.1°C) and stable normothermia from day 2.

Our real-life sample showed a male predominance of 83.5%, and the results should therefore be interpreted with caution regarding generalization to females. Furthermore, we are aware that TCD parameters may be influenced by some confounders that we have not adjusted for. In a smaller comparable study on OHCA patients, however, cerebral blood flow measured by Doppler ultrasound in patients with good and poor outcomes was not significantly influenced by heart rate, oxygen saturation, carbon dioxide partial pressure, pH value, blood levels of glucose, hemoglobin and sodium, or treatment with sedatives and catecholamines (22). In addition, a large, recent study has demonstrated that pH and CO2 levels after OHCA are not associated with differential outcomes (23). Even though our study sample is larger than most previous TCD studies on OHCA, we could, unfortunately, not have adjusted for all these parameters due to limited power. The same applies to further subgroup analyses, e.g., patients with different cardiac arrest etiologies. Our choice of reported TCD parameters reported may be criticized but was based on pre-decided, standard, flow-based parameters and included both systolic flow, mean flow, and diastolic flow as well as pulsatility index and resistance index for completeness. End diastolic flow (EDV) has been suggested to be important in other settings but comparison with studies that do not report complete flow parameters such as PSV is difficult and is beyond the scope of the present study. The Lindegaard index (mean velocity in MCA vs. mean velocity in ICA) has been suggested as an indicator of cerebral vasospasm in subarachnoid hemorrhage and could perhaps have been reported (24). However, as we did not, in advance, suspect intracranial vasospasm here, this index did not seem a prime target, and we suggest *post-hoc* addition of this and other parameters to be statistically unsound.

## Conclusion

With bedside TCD, we documented a distinct flow pattern after OHCA with an initial phase of hypoperfusion during TTM followed by a physiological hyperperfusion phase around day 3 and a sustained cerebral hyperperfusion up to days 5–7. This indicates irreversible brain tissue damage as demonstrated by the association with poor outcomes at 6 months. Patients with good outcomes had a normalization of flow around days 5–7.

## Data availability statement

The datasets presented in this article are not readily available due to ethical and data protection regulations in Norway, data access is restricted. However, limited tabulated anonymized data may be made available upon reasonable written request to the authors. Requests to access the datasets should be directed to CL, a.c.lundqvist@medisin.uio.no.

## Ethics statement

The studies involving humans were approved by Regional Ethics Committe of South-East Norway. The studies were conducted in accordance with the local legislation and institutional requirements. Written informed consent for participation in this study was provided by the participants' legal guardians/next of kin.

## Author contributions

EN, GA, KS, and CL planned and designed the overall NORCAST project. CL and AR designed the Neurological part of NORCAST with the TCD subproject. AR and LA collected the TCD data. CL, AR, JH, and LA did the neurological assessment of patients during the index hospitalization while CL and JH did the Neurological outcome assessment at 6 months. AR, CL, and JŠ analyzed the TCD data. EN, GA, KS, CL, AR, and JŠ interpreted the data. AR drafted the manuscript together with CL. All authors assessed, commented on, and approved the final version of the manuscript.
